# Early Measures of TBI Severity Poorly Predict Later Individual Impairment in a Rat Fluid Percussion Model

**DOI:** 10.3390/brainsci13091230

**Published:** 2023-08-23

**Authors:** Shelby M. Hetzer, Andrew Casagrande, Dima Qu’d, Nicholas Dobrozsi, Judy Bohnert, Victor Biguma, Nathan K. Evanson, Jennifer L. McGuire

**Affiliations:** 1 Neuroscience Graduate Program, University of Cincinnati, Cincinnati, OH 45267, USA; canslesy@mail.uc.edu; 2 College of Arts and Sciences Interdisciplinary Program—Neuroscience, University of Cincinnati, Cincinnati, OH 45221, USA; 3 Applied Pharmacology & Drug Toxicology Program, University of Cincinnati, Cincinnati, OH 45267, USA; 4 Department of Neurosurgery, University of Cincinnati College of Medicine, Cincinnati, OH 45267, USA; bohnerjy@ucmail.uc.edu (J.B.); kozioljl@ucmail.uc.edu (J.L.M.); 5 University of Cincinnati College of Medicine, Cincinnati, OH 45267, USA; 6 Division of Pediatric Rehabilitation Medicine, Cincinnati Children’s Hospital Medical Center, Cincinnati, OH 45229, USA; 7 Department of Pediatrics, University of Cincinnati, Cincinnati, OH 45229, USA; 8 Department of Neurology and Rehabilitation Medicine, University of Cincinnati, Cincinnati, OH 45267, USA

**Keywords:** traumatic brain injury (TBI), rats, early measures, neurologic severity score, fluid percussion injury

## Abstract

Background: Multiple measures of injury severity are suggested as common data elements in preclinical traumatic brain injury (TBI) research. The robustness of these measures in characterizing injury severity is unclear. In particular, it is not known how reliably they predict individual outcomes after experimental TBI. Methods: We assessed several commonly used measures of initial injury severity for their ability to predict chronic cognitive outcomes in a rat lateral fluid percussion (LFPI) model of TBI. At the time of injury, we assessed reflex righting time, neurologic severity scores, and 24 h weight loss. Sixty days after LFPI, we evaluated working memory using a spontaneous alternation T-maze task. Results: We found that righting time and weight loss had no correlation to chronic T-maze performance, while neurologic severity score correlated weakly. Discussion: Taken together, our results indicate that commonly used early measures of injury severity do not robustly predict longer-term outcomes. This finding parallels the uncertainty in predicting individual outcomes in TBI clinical populations.

## 1. Introduction

An estimated 5 million people in the United States struggle with long-term disability as a result of a traumatic brain injury (TBI) [1]. Even in injuries classified as ‘mild,’ approximately 20–50% of patients have symptoms that continue beyond one year that can become permanent [2]. Although this is a significant problem, there is weakness in the ability to predict long-term outcomes after TBI. The successful treatment of patients with chronic symptoms requires either identifying at-risk patients for more intensive early intervention or developing successful treatment strategies for entrenched TBI symptoms. This is a challenge for preclinical research and identifying reliable indices of injury severity and long-term deficits to enrich study populations at longer time points would be invaluable. Some pre-injury characteristics are known to associate with worse outcomes, such as psychological factors [3], psychiatric history [4], premorbid cognitive ability [5], and age at injury [6]. Generally, more severe injuries are associated with higher odds of long-term cognitive and behavioral deficits following TBI, but there remains uncertainty about what measures of initial TBI severity are important. Perhaps because of this, there are no models that adequately predict individual patient outcomes, even though a number of measures have been suggested [7,8].

In preclinical TBI models, there are similarly a number of measurements suggested to correspond to injury severity that are collected as part of the initiative for common data elements in TBI basic research [9]. Yet, there are no set guidelines for the classification of injury severity, and recommendations are subject to change with different preclinical TBI methodology (e.g., fluid percussion injury versus weight drop). Some of the most common assessments used include regaining reflexes (e.g., righting reflex time [RRT] or pinna reflex), weight loss, and motor/cognitive behavioral changes (e.g., the Neurological Severity Score [NSS]). Studies that include these parameters, however, tend not to report correlations with end-point measures [10]. These measures are rarely correlated to functional outcomes and, if such statistics are done, it is almost always between group means rather than between an individual’s injury severity measurements and final outcomes. To date, correlations between measurements of functional severity and individual behavioral deficits are limited to acute time-periods after injury [11,12]. Pressing needs in TBI research include the ability to study individual differences in recovery trajectory from a common baseline and an understanding of how chronic deficits are established and maintained. To do this, researchers must accurately define the initial injury and have a reasonable estimation of future impairments therefrom.

In this study, our goal was to determine which of the commonly used indices of injury severity used in preclinical research would predict intra-individual deficits at a chronic timepoint within a cohort of rats with experimental TBI. Using the lateral fluid percussion injury (LFPI) model, we tested whether RRT, NSS, open field test, and post-TBI changes in weight correspond to chronically impaired working memory in individual rats. RRT and weight change after LFPI showed no correlation to later T-maze performance. We found a negative correlation of NSS to memory deficits 8 weeks after injury. However, this correlation is likely not strong enough to be useful in inferring future cognitive impairment early after TBI.

## 2. Materials and Methods

### 2.1. Subjects

**Figure 1:**
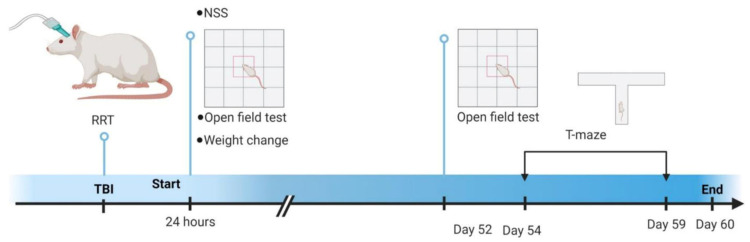
Timeline of experimental procedures. Initial injury severity measures were performed immediately after injury (RRT) or after 24 h (NSS, open field test, 24 h weight change). Delayed measures were conducted at Day 52 (open field test) and from Day 54 through 59 (T-maze spontaneous alternation). Experiments ended on Day 60. RRT—Righting Reflex Time; NSS—Neurological Severity Score. This figure was made using Biorender.

The animals for this study were 23 adult male Sprague-Dawley rats (275–300 g at craniotomy). The animals were housed in a climate-controlled vivarium on a 12:12 light dark cycle at the University of Cincinnati. The animals were pair housed when possible. All procedures described were reviewed and approved by the University of Cincinnati Institutional Animal Care and Use Committee. Figure 1 shows a timeline schematic of experimental procedures.

The animals in this study are pooled from 2 separate cohorts. A total of 11 of the 23 animals were fed a liquid diet for the final 3 days of the study, and the other 12 were given 200 ul of 50:50 sweetened condensed milk and water twice daily for 14 days. Prior to combining these data in the current analyses, we compared each experimental endpoint across the two cohorts, and found that there was no difference between the two cohorts, with the exception of the completion rate of T-maze trials (Figure S1). There was no correlation between completion rate and alternation percent, so this likely did not affect our experimental endpoints, as completion rate was not one of the endpoints examined. We also performed Monte Carlo permutation analysis on the cohorts using the R package MKinfer version 1.1 (https://cran.r-project.org/web/packages/MKinfer/index.html (accessed on 2 August 2023)). This approach did not change any of the statistical outcomes in the initial analysis.

### 2.2. Fluid Percussion

The fluid percussion procedure was performed as previously described [13,14]. We placed a craniotomy over the right parietal cortex, attached a conduit made from a modified 20 g syringe needle to the skull surface, and capped off the conduit to protect the brain surface. 72 h later the cap was removed, and the animal was anesthetized with 4% isoflurane for 5 min and attached directly to the lateral fluid percussion machine (Custom Design and Fabrication, Petersburg, VA, USA). The injury was created by forcing a pressure wave of sterile saline against the exposed brain surface (~2 atm pressure). The animals were weighed and monitored for recovery for one week after injury, then weekly thereafter.

### 2.3. Injury Appraisal

Reflex righting time was used as an immediate index of injury severity. After the TBI, the animal was placed on its back, and we recorded the time required to return to upright with all 4 feet on the ground. One day following LFPI, we performed an abbreviated neurological assessment [15].

### 2.4. Neurological Severity Score and Locomotor Activity

Prior to injury, the animals were trained for three days to walk a balance beam that was 3.8 cm wide and 91 cm long. The animals were trained until they could reliably cross without falling and voluntarily cross back and forth. One day after injury, we tested eye blink response in which a sterile swab was moved to almost touch the eye to elicit a response. Normal blink response on both sides received no points, one point was scored for a response on one side only, and two points were awarded if there was no response. We measured resistance to being dragged gently by the base of the tail, in which a normal response was extension of both forearms forward and reaching back with the hindlimbs when the bottom half the animal was lifted. No points were awarded for a normal response, one point was awarded if one side curled in towards the belly rather than extending, and two points were awarded if neither side extended. We tested sensory response by pinching the toes of the hind feet. If the limb was immediately jerked away, no points were scored. If the response was unilateral, one point was scored, and if there was no response on either side, 2 points were scored. We tested startle response in which the animal was allowed to wander on a table surface for 10–15 s, and a box of plastic pieces was dropped behind them. If they jumped, ran, or froze, no points were awarded. If they looked towards the sound or changed direction to move away from the sound, one point was scored, and if there was no response, two points were scored. Startle testing was conducted one animal at a time in a separate room from the other tests and was performed last. Balance was tested on the balance beam they had been trained on previously. Balance was scored on a four-point scale. Crossing the beam without slipping or turning around received zero points. One point each was scored if the animal slipped and almost fell but was able to recover, if they turned perpendicular to the beam to maintain balance, or if they turned around and failed to cross. If the animal fell from the beam, 4 points were scored. The animals were allowed four attempts, and the average of the four trials was used.

The day before craniotomy, rats were habituated to the open field arena, a round arena 63 cm in diameter divided into an inner circle and outer ring of equal area and evaluated for baseline activity and exploration. The rats were tested in the open field again one day after LFPI and then again 52 days after LFPI. The animals were tracked and recorded for 5 min in the field using Ethovision software version 11.5 (Noldus, Wageningen, The Netherlands). Data were scored for distance travelled. Two files were corrupted for baseline open field measurements, so analyses of presurgical behavioral data included only 21 animals.

### 2.5. T-Maze Alternation

Working memory was tested in a T-maze spontaneous alternation task from days 54–59 after injury [16]. In each trial, the rat was placed in the maze and allowed to freely choose the right or left arm. The rat was removed from the maze, the maze was cleaned, and after 30 s the rat was returned to the maze. The animals were tracked and recorded using Ethovision software, and animals were determined to have entered an arm when the center point of the animal’s body passed halfway down the arm and remained for at least 1s. The trial terminated when the animal entered the arm or after 2 min if no choice was made. The animals were given 10 trials per day for 6 consecutive days. T-maze data were scored for completion (completed trials/total trials × 100) and % alternation (alternations/completed trials × 100).

### 2.6. Statistical Analysis

Data was analyzed in Prism 9.0.2 (GraphPad, San Diego, CA, USA) using regression analyses to correlate initial measures of injury severity to later behavioral outcomes. Data were analyzed to determine normality (D’Agostino and Pearson), mean, range, and standard deviation for all measurements. Correlations were performed using Pearson’s r or Spearman’s rho respectively for normally and non-normally distributed data. Data are presented as range (minimum and maximum values), mean ± standard deviation, or as correlation coefficient (r), confidence interval, and *p*-value.

## 3. Results

### 3.1. Acute Injury Evaluation

**Figure 2:**
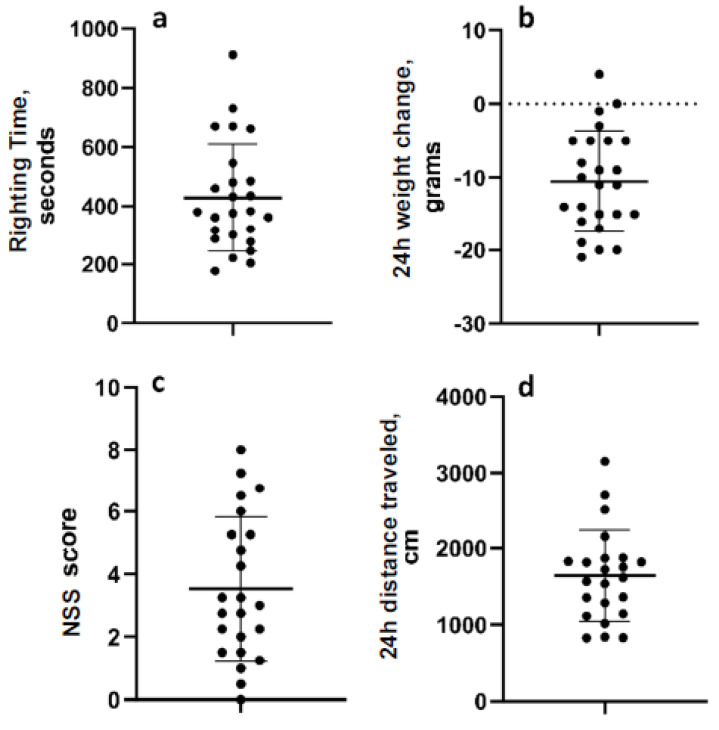
Acute measurements of injury severity. (**a**) Reflex righting time. (**b**) Weight change over the first 24 h after LFP. (**c**) Neurologic severity score 24 h after LFP. (**d**) Distance travelled in the open field 24 h after LFP. Data are mean ± SD.

The mean righting time for all 24 animals was 442.7 s (range 177–912 s; standard deviation 184.3 (Figure 2a). The mean change in weight overnight after LFPI was −11.87 g (range −21–0 g; standard deviation 6.05) (Figure 2b). In the neurological severity testing, the mean score was 3.53 (range 0–8; std. dev. 2.29), and the mean distance travelled in the open field one day after LFPI was 1639 cm (range 828.5–3156 cm; 595.6) (Figure 2c,d). Data were normally distributed. Post hoc analyses determined that there were no pre-injury or post-injury measurements that were significantly different between animals in the two experiments (Figure S1).

### 3.2. Chronic Behavioral Measurements

**Figure 3:**
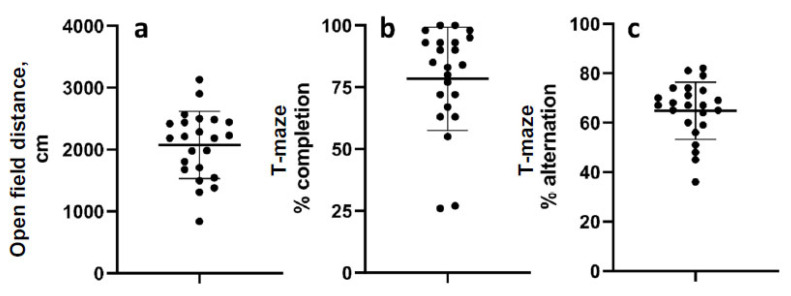
Chronic behavioral measurements. (**a**) Distance travelled in the open field 52 days after LFP. (**b**) Percentage of trials completed in the T-maze alternation test. (**c**) Percent alternations of completed trials in the T-maze test. Data are mean ± SD.

The mean distance travelled in the open field 52 d after LFPI was 2075 cm (range 839.5–3133 cm; standard deviation 542.5) (Figure 3a). The mean percentage of completed trials in the T-maze alternation task was 78.4% (range 26–100%, std. dev. 20.9), and the mean percent alternation was 65% (range 36–82% std. dev. 11.55) (Figure 3b,c). Data were normally distributed except for percent completion.

### 3.3. Correlation of Behavioral and Injury Severity Measurements to Percent Alternation

**Figure 4:**
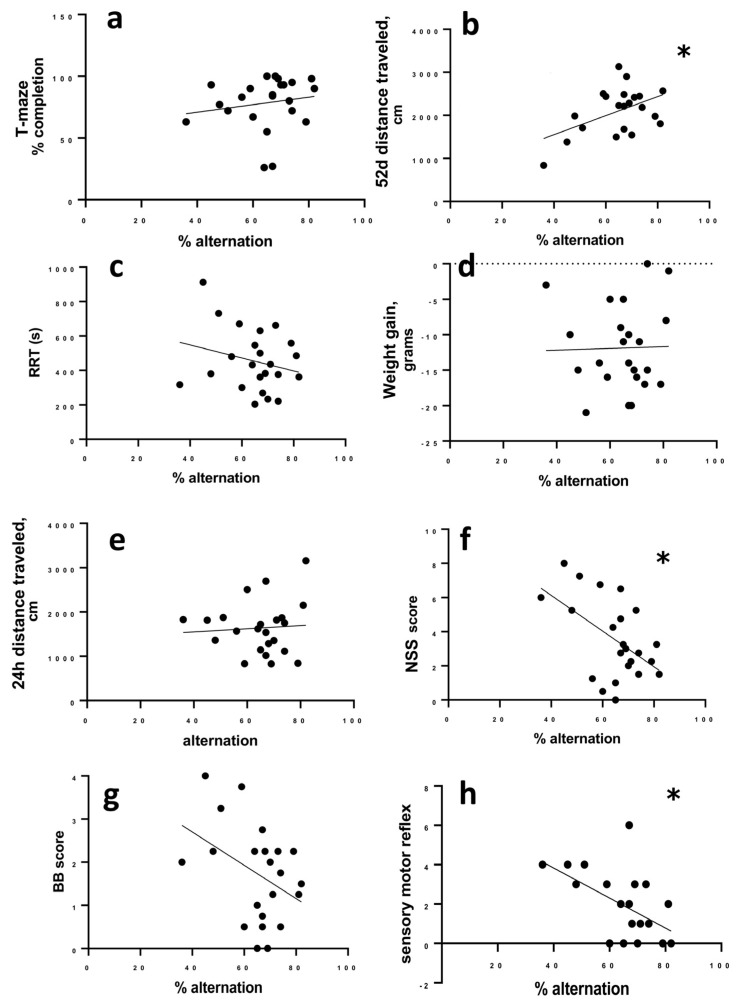
Correlation of alternation to other measures. (**a**) Completion of T-maze trials does not correlate to successful alternation. (**b**) At 52 days post-LFP, more active animals are more successful in the alternation task. (**c**) RRT, (**d**) change in weight, and (**e**) initial (24 h) activity do not correlate to successful alternation in the T-maze. (**f**) Animals with more impairments measured by NSS 24 h after LFP were more likely to have lower alternation success. This correlation of NSS to alternation was not significant for balance beam performance (**g**), but was significantly correlated to sensorimotor measures (**h**). Data were analyzed by Spearman’s rho (completion to alternation) or Pearson’s r (all other data). Significance was determined by *p* > 0.05 when r deviated from zero; * *p* < 0.05.

The percentage of successful alternations did not correlate to the percent of completed trials in the T-maze (r = 0.2985; 95% CI −0.1423 to 0.6406, *p* = 0.1665) (Figure 4a), but there was a significant correlation between alternation and activity in the open field on day 52 post LFPI, as more active animals alternated more successfully (r = 0.509; 95% CI 0.1220 to 0.7613; *p* = 0.013) (Figure 4b).

Percent alternation did not correlate to RRT (r = −0.2440; 95% CI −0.5614 to 0.2614, *p* = 0.263), overnight weight loss after LFPI (r = 0.024; 95% CI −0.4127 to 0.4330; *p* = 0.914), or distance travelled in the open field 24 h after LFPI (r = −0.0550, 95% CI −0.4662 to 0.3775, *p* = 0.8068) (Figure 4c–e). We did find a significant correlation between the NSS score to alternation where animals with higher NSS scores (more initial deficit) were more likely to have lower alternation rates (r = −0.526; −0.7711 to −0.1455; *p* = 0.01) (Figure 4f).

To determine whether there were specific components of the NSS that were more strongly associated with chronic T-maze performance, we looked at the two main components of the NSS, sensory reflex (8 points of the total NSS score) and balance beam (4 points of the total NSS score) separately. Balance deficits scored on the balance beam were not significantly correlated to T-maze alternation (r = −0.378; 95% confidence interval −0.6834 to 0.04102; *p* = 0.0757) (Figure 4g). Deficits in sensory reflexes correlated to later T-maze alternation with animals having greater sensory impairment being more likely to have lower alternation (r = −0.466; 95% CI −0.7365 to −0.06626; *p* = 0.025).

## 4. Discussion

In the current study, our goal was to determine whether current measures of injury severity used in preclinical research predicted individual chronic working memory deficits. In particular, we were focused on the ability to perform within-individual analysis and prediction within a group of injured animals, rather than attempting to compare injured to control rats. We utilized several acute measures of severity and correlated them to individual longer-term measure of cognitive performance. As early measures of TBI severity, we measured RRT, early weight change after injury, NNS, and locomotor behavior in an open field. As a measure of cognitive performance, we used spontaneous alternation in a T-maze as a measure of working memory. We found that only NSS score significantly correlated with delayed spontaneous alternation behavior, and this only weakly. Specifically, only the sensorimotor tests within the NSS had a statistically significant correlation with later cognitive performance. These results suggest that none of these early measures provides robust predictive ability of chronic TBI outcomes in this model.

### 4.1. Reflex Righting Time

The initial use of the righting reflex is based in sound human TBI research demonstrating correlations between loss of consciousness and injury severity [17,18]. In animal studies, RRT is presumed to resemble the human’s regaining of consciousness as the rodent rights itself from a resting position into an active standing/walking orientation and is used as a surrogate for loss of consciousness [19]. RRT is frequently used for initial assessments as it is easily measured. Many labs, including ours, have reported RRT as the sole measurement of injury severity. In cases where study endpoints are close to the time of injury, RRT may be the only available behavioral index of injury [20]. However, there are no data on RRT’s predictive capacity for long-term deficits. A moderate correlation exists between magnitude of injury and RRT [21]. In a weight drop injury model, RRT correlated to degree of tissue degeneration at acute times (up to 7 days post-injury) [22] and in another weight drop study, mice with longer RRT had worse behavioral performance on an avoidance task [23]. However, this is not demonstrated across all models, and immediate post-traumatic seizure was associated with shorter RRT in a rat fluid percussion model (Rowe et al., 2018 [24]). Overall, RRT does not appear as useful for predicting functional outcomes other than perhaps lesion volume [25].

In patients, coma or unconsciousness is used in classifying initial severity of TBIs, usually via the Glasgow Coma Scale [26]. In general, length of coma after injury in human populations correlates with degree of diffuse axonal injury [27] and worse neuropsychological outcomes [28]. More specifically, coma lengths greater than 4 weeks correlate with poor long-term outcomes [29]. Despite length of coma having reasonable usefulness in severe TBI patients, we found that RRT is not a sensitive predictor of longer-term cognitive outcomes for individuals in our TBI model. A possible reason for this difference between human and animal studies is that human patients with long coma likely have more severe injuries than what is survivable in animal studies. Severe TBIs with high mortality rates has not been extensively studied in animal models [30], and although mechanical ventilation alone significantly improves mortality in animal models of more severe injuries (e.g., [31]), it is not routinely used in animal studies.

### 4.2. Change in Weight after TBI

Change in weight after TBI is reported less consistently and as an adjunct to other measures of injury severity. Percentage of weight loss has been argued to correspond to injury severity in the FPI model, but supporting evidence was limited [32]. However, weight loss over the first 4 days after lateral fluid percussion injury is a better (albeit weak) predictor of delayed spontaneous seizure development than behavioral assessments [33]. Although intuitively it makes sense that animals with worse injuries may feel worse, eat and drink less, and lose weight, our data fail to support early weight changes as a predictor of long-term outcomes.

### 4.3. Neurological Severity Score

Although variations of the neurological severity score (NSS) are often implemented as a standard assay for evaluating injury severity, there is minimal evidence demonstrating that early behavioral deficits in NSS correlate to future outcomes within individuals. Several groups have asserted the high “predictive” power of their measurements (e.g., [34,35]). However, we found no study that analyzed severity scores in relation to other behavioral or functional outcomes. Instead, these studies compared early NSS scores with later scores but did not use them as a predictive tool for other endpoints. While NSS showed the strongest correlation to chronic T-maze performance in our data, other groups have found no correlations to other long-term functional outcomes. For example, Huynh, et al. [36] showed no differences in NSS between sham and TBI mice, yet there were clear impairments in Morris Water Maze performance in TBI mice. There is also little to no support for correlations between righting time and NSS [37]. Poor NSS performance correlated to more prolonged post-impact seizure-like behavior, but there was no correlation between NSS and the development of longer-term post-traumatic epilepsy [33]. Several studies have correlated NSS with histological and imaging analyses of injury severity, such as gross lesion volumes [38], glial activation [39,40,41], and blood levels of cell-free DNA [42]. In our hands, NSS was most predictive of later intra-individual T-maze impairment, although the finding was not robust. In combination, the data suggest that NSS is perhaps the best available measure to stratify injury, but there is enough inconsistency to warrant caution.

### 4.4. Open Field Test

We used the open field test as an early and chronic measure of locomotor activity, measuring the total distance travelled. The open field test is considered to be the most standardized test for general locomotor function [43]. This test was an important control for the spontaneous alternation task, since locomotor activity is necessary for rats to be able to perform the spontaneous alternation choices. We saw a correlation between chronic performance in this task and chronic T-maze performance, wherein higher locomotor activity positively correlated with increased spontaneous alternation. This result may suggest that locomotor activity and exploratory behavior leads to more engagement in the T-maze task. In other words, more active animals appear to be more likely to complete the T-maze task.

### 4.5. Spontaneous Alternation Task

The spontaneous alternation task takes advantage of a rat’s natural foraging behavior, and is considered to be a measure of working memory function [16]. Normal performance of this task requires proper function in the hippocampus and prefrontal cortex [44], and has an excellent ability to detect hippocampal dysfunction [45,46]. We chose this behavior as a chronic end point due to its utility as a measure of working memory and hippocampal function, because working memory deficits are a common chronic cognitive impairment in human TBI patients [47,48,49]. We have previously found that there are significant deficits in this behavior at chronic time points after lateral fluid percussion injury [13], consistent with chronically injured humans.

### 4.6. Clinical Prediction Tools

Our findings, although frustrating, are consistent with a relatively low rate of prognostic success in the clinical literature. The most widely used measure of initial injury severity is the Glasgow Coma Scale (GCS) [26]. Other common means of differentiating between milder and more severe injuries include length of post-traumatic amnesia and duration of loss of consciousness. In more severe injuries, these measures, when combined with other diagnostic criteria like age or other scoring modalities like the Full Outline of UnResponsiveness (FOUR) score [50], can be useful when making broad generalizations of recovery potential. However, the GCS and FOUR scores are predominantly used to predict mortality from severe injuries or acute recovery before discharge from the hospital [51,52].

In the case of more mild injuries, such as concussion, attempts to predict chronic outcomes have been largely unsatisfactory [53]. This is an important failure, because although most patients with concussions are expected to recover completely, a significant minority (around 10–20%) have post-concussion symptoms that persist beyond 3 months, and a large number of these patients may go on to have chronic symptoms [54]. Several recent studies have argued that tests of vestibular/ocular motor function (e.g., smooth pursuit) [55] or assessments determined with the Immediate Postconcussion Assessment and Cognitive Test (ImPACT), including visual motor deficits [56], may predict symptom duration, but they were not evaluated further than the first two weeks after injury rather than the 1–3 months required for a post-concussive syndrome diagnosis [57]. Serum interleukin 6 (IL-6), may provide a potential biomarker of post-concussion symptoms, but was only followed to 45 days post injury [58]. So far, the only factor that appears to predict chronic (12 month) outcomes is symptoms persisting a few months after injury; this is associated with worse self-reported cognitive and emotional outcomes [59]. In summary, the literature suggests that while acute markers of long-term outcomes would help with treatment planning and managing patient expectations after TBI, these measures are lacking.

### 4.7. Limitations of This Study

In our model, as with virtually all preclinical TBI models, the TBI is performed under anesthesia. The isoflurane used in these studies may have some neuroprotective effects, and may also affect measures like the RRT [60]. Our only chronic behavioral outcome was T-maze alternation. It is conceivable that early measures of TBI severity correlate better to other behavioral endpoints such as spatial learning, anxiety, or conditioned responses. We elected to perform a single test to eliminate the confounding of experiential learning on the interpretation of subsequent behavioral tests and the relationship of TBI severity measures to later tasks. T-maze was selected because it relies on working memory (deficits in working memory and other executive functions are frequent symptoms in chronic injury) and because it measures an instinctive behavior and thus does not require training, which could further confound the analysis.

In the original animal cohorts, we included naïve controls. However, we only included rats with TBI in the current analysis, because we were only interested in the ability of early measures of injury severity to predict long-term outcomes in TBI. Because we used a within-subjects correlation analysis, we felt that including controls would not add to the findings of this study. In addition, the experimental animals used in this study were pooled from several cohorts, in order to increase the number of animals analyzed and thus increase the chance of finding significant correlations between early measures and long-term outcomes. Although the cohorts were mostly treated identically, there were two diet conditions used between these animals. As noted in Section 2.1, we compared two diet conditions to each other. For the experimental outcomes used in this study there were no differences between the cohorts, although there was a difference in completed T-maze trials (See Figure S1). We then re-analyzed these results using a Monte Carlo Permutation analysis, to minimize the effect of random variations in the data, and the results were not significantly changed. Completed T-maze trials did not correlate to alternation percentage. Thus, it is not likely that this had a significant effect on our results. Finally, the work reported only used a single percussion pressure. Futures studies could build on this work by adding additional fluid percussion pressures to add a variety of injury severities to the analysis.

## 5. Conclusions

Although preclinical TBI models allow for the control of injury characteristics, current measures of initial injury severity do not closely correlate with individual long-term TBI outcome. Although there was some correlation between the NSS and longer-term T-maze performance, the correlation is weak, and thus is not satisfactory to predict deficits for any individual. Taken together, our results suggest that, at least in rat lateral fluid percussion, improved early measures of injury severity are needed.

## Data Availability

The data in this study are available upon request from the corresponding author.

## Supplementary Materials

The following supporting information can be downloaded at: https://www.mdpi.com/article/10.3390/brainsci13091230/s1, Figure S1: Cohort comparison, Table S1: Monte Carlo Permutation analysis of data from experiments 1 and 2. Results are 1000 permutations of Welch 2-sample *t*-test. Data were generated in R using the perm.t.test function in the Package mKinfer. There are no significant differences in results between those shown in Figure S1 and those found using Monte Carlo Permutation analysis.
