# Supplementary material for: Early Measures of TBI Severity Poorly Predict Later Individual Impairment in a Rat Fluid Percussion Model
Source: Brain Sci. 2023 Aug 23;13(9):1230. doi: 10.3390/brainsci13091230 (PMC10526292; doi:10.3390/brainsci13091230)
Supplement: Supplementary file 1 [file brainsci-13-01230-s001.zip › brainsci-2519879-supplementary.pdf]

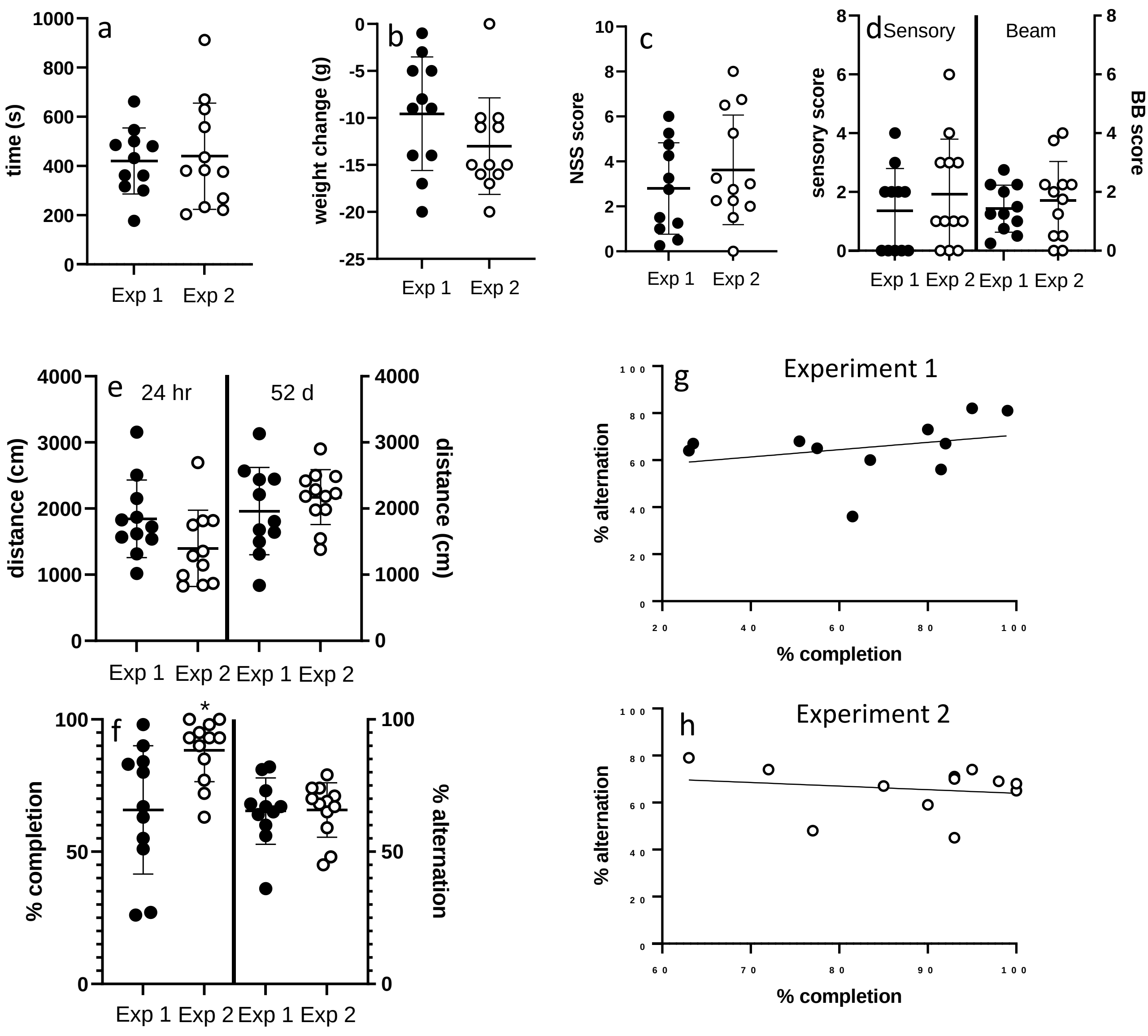

Figure S1. Comparison between Experiment 1 and Experiment 2 for the parameters reported. We found no difference between experiment 1 and experiment 2 in a) righting time, b) weight change, c) total NSS score, d) sensory or motor components of the NSS, and e) distance traveled in the open field either 24 hours or 52 days after TBI, f) we found Experiment 1 animals completed a lower percentage of spontaneous alternation trials ($p = 0.0213$, but no difference in % alternation in completed trials. g) In experiment 1, there was no significant correlation between the percentage of trials completed and % alternation ($p = 0.369$, $R^2 = 0.090$. h) In experiment 2 there was no significant correlation between the percentage of trials completed and % alternation ($p = 0.5868$, $R^2 = 0.0306$. Data were normally distributed. N=11 experiment 1, n=12 experiment 2. Data were analyzed using Welch 2-sample t-test with no correction for multiple comparisons. * = $p < 0.05$. Data are mean +/- Standard deviation or Pearson's correlation.

Test		p-value	95% Confidence Interval	p< 0.05
NSS	Exp 1 vs Exp 2	0.8449	-1.8358-3.5318	
	Monte Carlo permutation	0.8475	-1.6609-2.0417	
RTT	Exp 1 vs Exp 2	0.6806	-188.05-125.46	
	Monte Carlo permutation	0.6823	-177.59-112.36	
Weight change	Exp 1 vs Exp 2	0.3637	-7.5436-2.5909	
	Monte Carlo permutation	0.3689	-7.747-3.0199	
sensory	Exp 1 vs Exp 2	0.7944	-1.3033-1.6821	
	Monte Carlo permutation	0.7412	-1.1893-1.5984	
Beam	Exp 1 vs Exp 2	0.9936	-0.9656-0.99732	
	Monte Carlo permutation	0.9705	-0.9072-0.9223	
Open field 24h	Original analysis	0.0733	-941.81-46.256	
	Monte Carlo permutation	0.0731	-948.20-27.016	
Open field 52d	Original analysis	0.3865	-289.79-696.20	
	Monte Carlo permutation	0.3784	-239.72-642.10	
Completion %	Original analysis	0.0213	3.529-37.516	*
	Monte Carlo permutation	0.0142	3.7576-37.516	*
Alternation %	Original analysis	0.7400	-8.2074-11.374	
	Monte Carlo permutation	0.7239	-7.1667-10.1667	

Table S1. Monte Carlo Permutation analysis of data from experiments 1 and 2. Results are 1000 permutations of Welch 2-sample t-test. Data were generated in R using the perm.t.test function in the Package mKinfer. There are no significant differences in results between those shown in Fig. S1 and those found using Monte Carlo Permutation analysis.
